# Supplementary material for: Post-transcriptional cross- and auto-regulation buffer expression of the human RNA helicases DDX3X and DDX3Y
Source: Genome Res. 2025 Jan;35(1):20–30. doi: 10.1101/gr.279707.124 (PMC11789639; doi:10.1101/gr.279707.124)
Supplement: Supplement 2 [file Supplemental_Figures.pdf]

SUPPLEMENTAL Figures

Post-transcriptional cross- and auto-regulation buffer expression of the human RNA helicases *DDX3X* and *DDX3Y*

Shruthi Rengarajan, Jason Derks, Daniel W. Bellott, Nikolai Slavov, David C. Page

SUPPLEMENTAL FIGURES

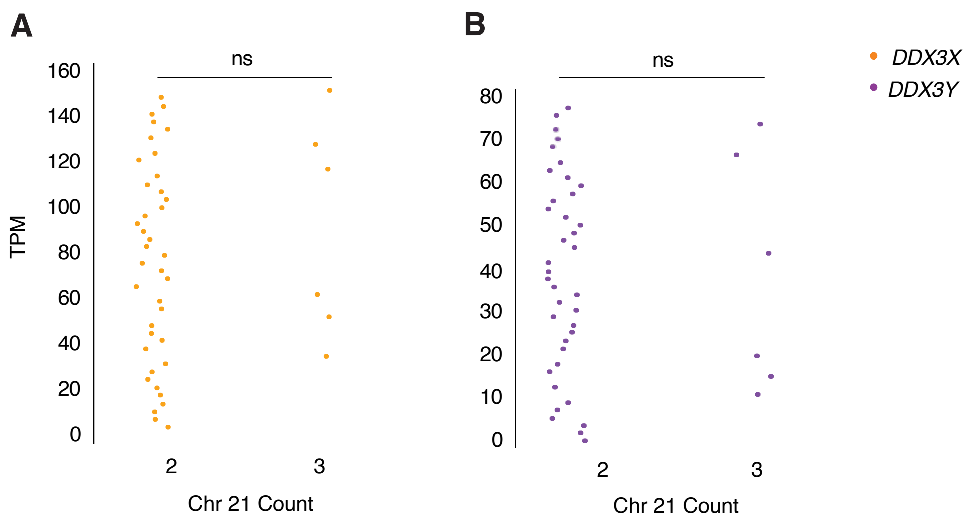

Supplemental Figure S1: Effects of Chromosome 21 copy number on *DDX3X* and *DDX3Y* expression. Each point represents a lymphoblastoid cell line from one XX or XY individual with either two or three copies of Chr 21 demonstrating that A) *DDX3X* and B) *DDX3Y* levels are unaffected by trisomy 21. Statistical significance determined by Mann-Whitney *U* test.

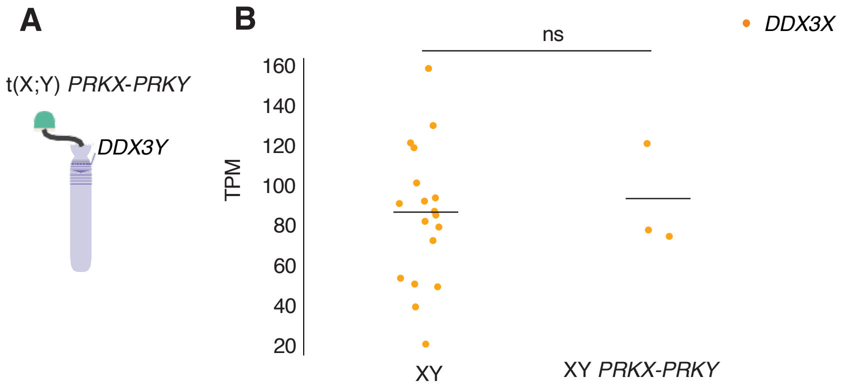

Supplemental Figure S2: *DDX3X* levels in Chromosome Y abnormalities that do not alter *DDX3Y*. A) Abnormal recombination between *PRKX* and *PRKY* results in X-Y translocation and partial deletion of the Y chromosome that leaves *DDX3Y* intact. B) LCLs derived from individuals with *PRKX-PRKY* translocations have the same levels of *DDX3X* transcripts as 46,XY individuals. Statistical significance determined by Mann Whitney - U test.

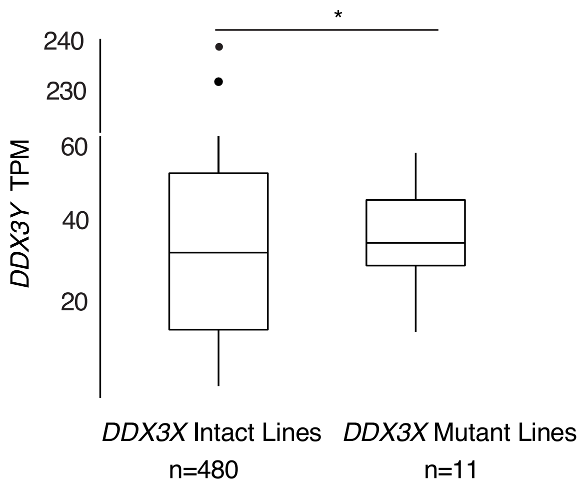

Supplemental Figure S3: Analysis of *DDX3Y* levels in Cancer Cell Line Encyclopedia dataset. *DDX3Y* transcripts are more abundant in XY cancer cell lines with damaging *DDX3X* mutations. Statistical significance determined by one tailed *t*-test, **$p < 0.05$

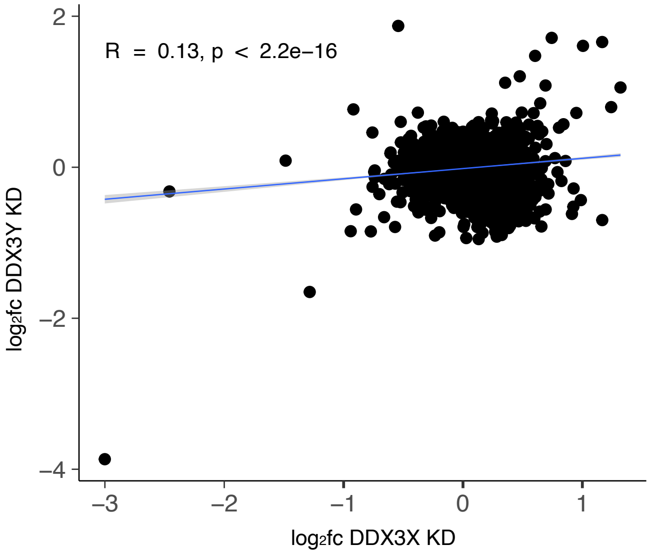

Supplemental Figure S4: Genome-wide transcriptional effects of *DDX3X* and *DDX3Y* knockdown. Scatterplot showing $\log_2$ fold change value of both knockdowns; each point represents an expressed gene. R values and statistical significance calculated using Pearson's correlation.

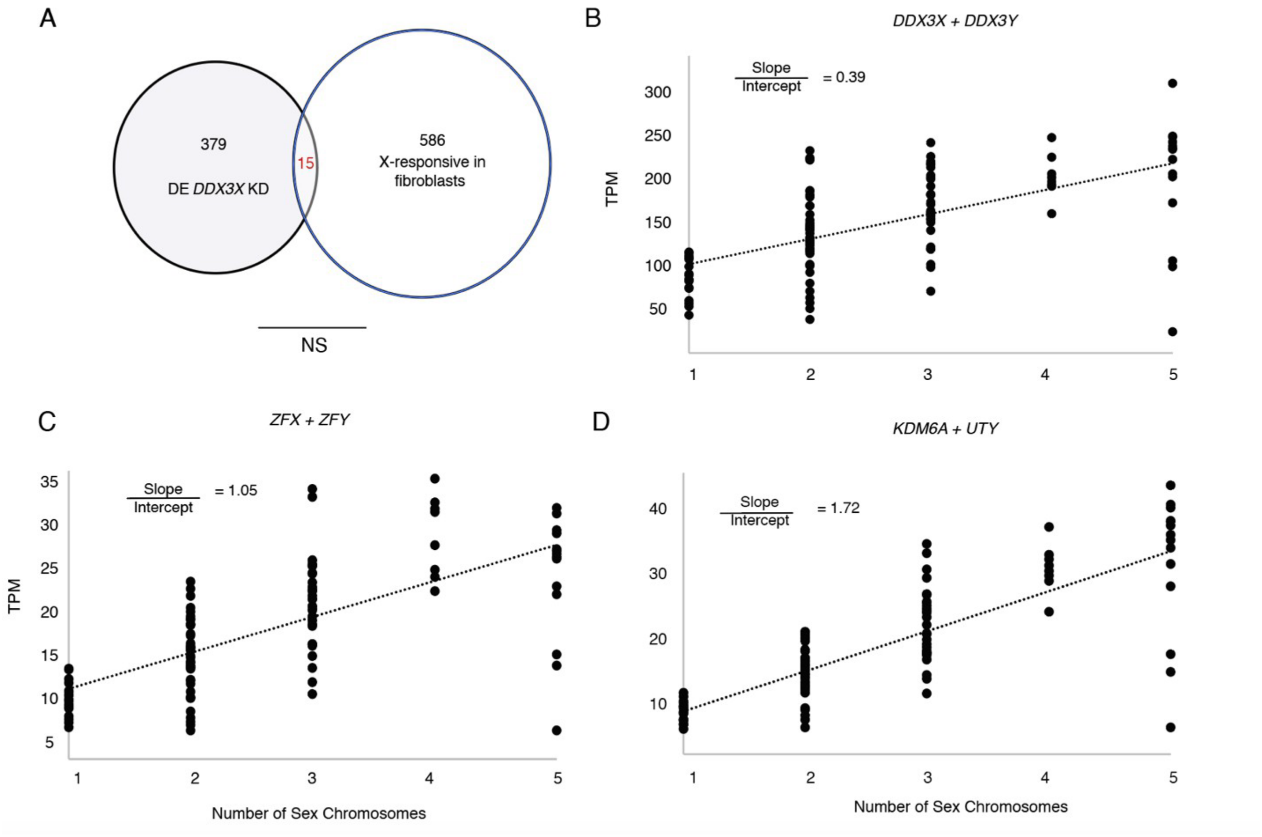

Supplemental Figure S5: Contribution of *DDX3X* and *DDX3Y* to aneuploidy gene expression. A) There is no overlap between genes that are differentially expressed upon *DDX3X* KD in XY fibroblasts and those that respond to X-chromosome dosage in fibroblasts with X chromosome aneuploidies. Statistical significance assessed using hypergeometric test. B) The increase in the total expression of *DDX3X* + *DDX3Y* with sex chromosome copy number is buffered compared to similarly constrained X-Y pair genes *ZFX*/*ZFY* (C) and *KDM6A*/*UTY* (D).

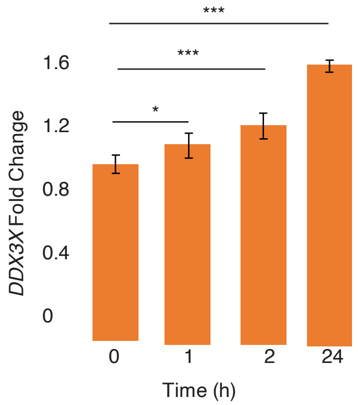

Supplemental Figure S6: Treatment of XX cells with *DDX3X* inhibitor RK-33. *DDX3X* transcript levels (by qPCR) in 46,XX fibroblasts are significantly elevated when treated with 2 μM RK-33 (inhibitor of DDX3X helicase activity), proportional to duration of treatment. Statistical significance determined by one-sided *t*-test on delta Ct values. Error bars indicate standard deviation of three technical replicates. * $p < 0.05$, *** $p < 0.001$.

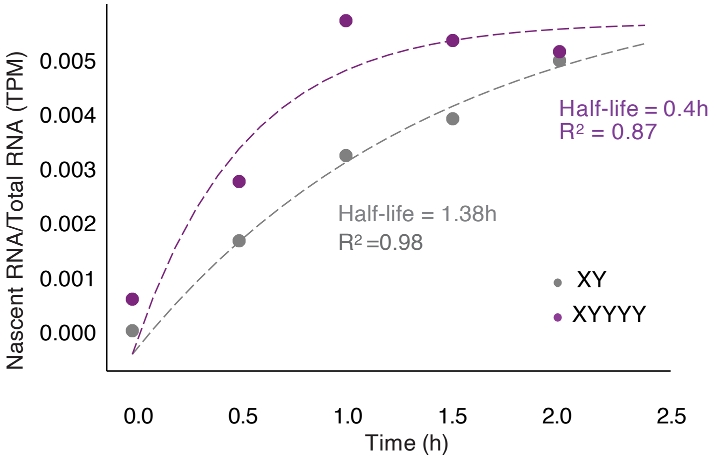

Supplemental Figure S7: Long time course metabolic labeling of XY and XYYYY cells. A) *DDX3X* mRNA half-life is 0.4h in XYYYY LCLs compared to 1.38h in a 2 hr independent metabolic labeling experiment. Normalized fraction of nascent/total *DDX3X* mRNA were fit to the equation y = α/β × 1-eβ/t to obtain β (half-life).B) *DDX3Y* levels are elevated and *DDX3X* levels decreased in 49,XYYYY LCLs compared to 46,XY LCLs.
